# Sub-national TB prevalence surveys in India, 2006-2012: Results of uniformly conducted data analysis

**DOI:** 10.1371/journal.pone.0212264

**Published:** 2019-02-22

**Authors:** V. K. Chadha, Sharda M. Anjinappa, Paresh Dave, Kiran Rade, D. Baskaran, P. Narang, C. Kolappan, K. Katoch, S. K. Sharma, V. G. Rao, A. N. Aggarwal, P. Praseeja, R. Jitendra, S. Swaminathan

**Affiliations:** 1 National Tuberculosis Institute, Bangalore, Karnataka, India; 2 Central Leprosy Teaching and Research Institute, Chenglepattu, Tamil Nadu, India; 3 State TB Cell, Gujarat, Ahmedabad, India; 4 Central TB Division, Ministry of Health & Family Welfare, New Delhi, India; 5 World Health Organization, country office for India, New Delhi, India; 6 National Institute for Research in Tuberculosis, Chennai, India; 7 Department of Microbiology, Mahatma Gandhi Institute of Medical Sciences, Sewagram, Wardha, Maharashtra, India; 8 National JALMA Institute for Leprosy and Other Mycobacterial Diseases, Agra, Uttar Pradesh, India; 9 Department of Medicine, All India Institute of Medical Sciences, New Delhi, India; 10 Regional Medical Research Centre for Tribals, Indian Council of Medical Research, Jabalpur, Madhya Pradesh, India; 11 Department of Pulmonary Medicine, Postgraduate Institute of Medical Education and Research, Chandigarh, India; 12 The Indian Council of Medical Research (ICMR), New Delhi, India; 13 World Health Organization, Geneva, Switzerland; Indian Institute of Science, INDIA

## Abstract

**Setting:**

Community based tuberculosis (TB) prevalence surveys in ten sites across India during 2006–2012

**Objective:**

To re-analyze data of recent sub-national surveys using uniform statistical methods and obtain a pooled national level estimate of prevalence of TB.

**Methods:**

Individuals ≥15 years old were screened by interview for symptoms suggestive of Pulmonary TB (PTB) and history of anti-TB treatment; additional screening by chest radiography was undertaken in five sites. Two sputum specimens were examined by smear and culture among Screen-positives. Prevalence in each site was estimated after imputing missing values to correct for bias introduced by incompleteness of data. In five sites, prevalence was corrected for non-screening by radiography. Pooled prevalence of bacteriologically positive PTB was estimated using Random Effects Model after excluding data from one site. Overall prevalence of TB (all ages, all types) was estimated by adjusting for extra-pulmonary TB and Pediatric TB.

**Results:**

Of 769290 individuals registered, 715989 were screened by interview and 294532 also by radiography. Sputum specimen were examined from 50 852 individuals. Estimated prevalence of smear positive, culture positive and bacteriologically positive PTB varied between 108.4–428.1, 147.9–429.8 and 170.8–528.4 per 100000 populations in different sites. Pooled estimate of prevalence of bacteriologically positive PTB was 350.0 (260.7, 439.0). Overall prevalence of TB was estimated at 300.7 (223.7–377.5) in 2009, the mid-year of surveys. Prevalence was significantly higher in rural compared to urban areas.

**Conclusion:**

TB burden continues to be high in India suggesting further strengthening of TB control activities.

## Introduction

Tuberculosis (TB) has been known to be one of the major public health problems and India continues to be the highest TB burden country accounting for 2.8 million incidence cases in 2015 out of 10.4 million cases globally [1]. World Health Organization (WHO) has derived this incidence from the prevalence as obtained in Gujarat where a state level survey was carried out during 2011–12 assuming that the prevalence in the state being more developed economically is unlikely to be higher than the national average. TB specific mortality has been estimated at 480 000 using the data from Vital Registration and verbal autopsy studies in limited geographical areas [1, 2]. Direct estimates of prevalence, for the first time in the country, were obtained through a nationwide prevalence survey in 1955–58 [3]. Subsequently, a large number of district/sub-district level surveys conducted by various investigators revealed that TB continued to be a high burden disease in India and there was wide variation in prevalence across different parts of the country [4, 5]. In the year 2005, Government of India (GOI) took a decision to conduct prevalence surveys in eight conveniently located sites in the country using a generic protocol [6–12]. Subsequently, two more surveys have been undertaken- one state level survey and another in a south Indian metropolitan city [13, 14]. The published results from five of these ten sites did not account for non-screening by chest radiography and data from some of the sites was analyzed differently. In this manuscript, we re-present the results of uniformly conducted data analysis across all sites, also correcting for non-screening by radiography using the statistical methods recommended in WHO guideline [15]. Thus estimated prevalence from all the sites have been pooled to obtain a national level estimate of bacteriologically positive pulmonary TB in adults and then the overall prevalence of TB (all types) in the community after adjusting for extra pulmonary TB and TB in children.

## Material and methods

### Study settings

Surveys have been carried out in ten sites across eight states in different parts of the country- one was a state level survey, seven district level and two sub-district level. Gujarat where state level survey was carried out is situated in the West Coast of India, two sites (Sahibzada Ajit Singh Nagar {SAS Nagar}, Faridabad) were located in the north, two (Jabalpur, Wardha) in central India, two (Kanpur, Banda) in the east and three (Chennai, a sub-division of Thiruvallur district, Nelamangala sub-division of rural Bangalore district) in the south. Background information of survey sites is given in Table 1. Altogether about 78 million people resided in the geographical jurisdictions of these survey sites; population size in individual sites varied between 0.3–60.4 million. The entire population in Nelamangala was rural while it was all urban in Chennai with 19% living in slums. The remaining sites had a mix with proportion of urban population varying between 10–67%.

**Table 1:** Sub-national tuberculosis prevalence surveys in India, 2006–2012—Background information of survey sites.

Site	Survey period	Population* (million)	Proportion (%) of population living in Urban areas	Year of RNTCP implementation [16]	Case notification rate in the mid-year of survey^£^^,^^β^			Treatment success rate in NSP cases [16]^Ω^
					All TB [16]	NSP [16]	Previously treated smear positive [16]	
A Sub division of Thiruvallur district	2006–08	0.5	10	1999	146	54	20	82
(Tamil Nadu)^@^								
Wardha, (Maharashtra)^$^	2007–09	1.3	30	2002	106	48	14	89
Nelamangala sub-division of Bangalore Rural district, (Karnataka)^@^	2008–10	0.3	0	2002	120	51	13	80
Chennai city,	2010–12	4.7	100	2001	142	50	20	87
(Tamil Nadu)^$^								
Gujarat^^^	2011–12	60.4	43	Expanded in phases from 1997 to cover entire state in 2006	124	59	36	88
Faridabad (Haryana)^$^	2008–09	2.2	56	2000	166	49	25	85
Kanpur		4.8	67	2004	156	30	29	79
(Uttar Pradesh)^$^	2008–10							
Banda,		1.8	16	2005	117	44	32	87
(Uttar Pradesh)^$^								
Jabalpur,	2009–10	2.5	57	2003	134	50	19	72
(Madhya Pradesh)^$^								
SAS Nagar, (Punjab)^$^	2008–09	0.7	39	2006	178	66	27	83

*For the year of initiating survey; Geographical coverage:

^@^sub-district level

^$^district-level

^^^state-level; texts in brackets are the names of the State

^£^registered for treatment

^β^Figures given for Thiruvallur and Nelamangla are for the respective districts

^Ω^registered in the year previous to the year for which notification rates are given in the previous column

NSP: New Smear Positive; RNTCP: Revised National TB Control Program

DOTS, an internationally recommended strategy for control of TB was adopted by India in the form of the Revised National TB Control Program (RNTCP) from 1997 and expanded in phases to cover the entire country by 2006. The year of its implementation in respective sites is given in Table 1. Total TB (all age-groups, all types) case notification rates in the mid-year of the survey varied between 106–178 per 100,000 populations across the survey sites; notification rates of new smear positive (NSP) cases between 30–66. SAS Nagar reported the highest all TB as well as NSP notification rates, Wardha had the lowest all TB notification rate while Kanpur reported the lowest NSP rate. In half of the sites, treatment success rate among NSP cases was below 85%, the then target under RNTCP and was as low as 72% in Jabalpur. [16] (Table 1). Treatment success rate among previously treated smear positive PTB cases in Chennai and Gujarat was 61% and 67% respectively and not available for other sites for the respective years.

### Sample size estimation

For sample size estimation, except for Gujarat and Thiruvallur, the expected prevalence (P) of bacteriologically positive Pulmonary TB (PTB) in adults was arbitrarily considered at 400 per 100,000 population assuming it to be similar to the prevalence estimated during nationwide survey in 1950s [3]. However, since screening by X-ray could not be deployed in 5 sites, sample size in these sites was later revised upwards, considering the value of P at 240 per 100,000 assuming that 40% of the prevalent cases in the survey population would be missed. In Gujarat, the expected prevalence of bacteriologically positive PTB was considered at 190 based on the observed smear positive notification rate under RNTCP and assuming equal number of cases outside the program. In Thiruvallur, the sample size for each of the serial surveys was estimated to find out the incidence, expected at 260 per 100000 in the intervening period [17]. For each of the sites, sample size was calculated to estimate the prevalence within 20% of the true value at 5% level of significance, design effect of 2 to account for cluster sampling and the non-response rate at 10%. The estimated sample size thus estimated for different sites and sampling methods are presented in Table 2.

**Table 2:** Estimated sample size, sampling technique, screening tools, eligibility for sputum collection and method of sputum smear examination, by site.

Site	Estimated n	Sampling technique	No. of clusters	Screening tools	Criteria for eligibility of sputum collection based on screening	Method of sputum smear examination
**Sub division of Thiruvallur district**	82000	Simple Random Sampling of clusters stratified by Rural /Urban[Rural cluster = village, Urban cluster = municipality/corporation/cantonment/notified town area]	53 [R = 50, U = 3]	Interview, MMR	i.Symptoms present ii.Previously diagnosed cases in earlier survey iii.Chest radiograph showing any pulmonary abnormality, TI	FM
**Wardha**	47828	PPS sampling of clusters stratified by Rural /Urban [Rural cluster = village, Urban cluster = Urban ward], further stratified by population size	54 [R = 45, U = 9]	Interview, MMR	i.Symptoms present ii.History of ATT iii.Chest radiograph showing any pulmonary abnormality, TI	Conventional^$^
**Nelamangala, Bangalore Rural district**	68400^#^	Simple Random sampling of clusters [group of villages in the jurisdiction of a Gram panchayat]	15 [All Rural]	Interview, MMR		FM
**Chennai city**	53142	PPS sampling of clusters [urban ward]	62 [All Urban]	Interview, MMR		FM
**Gujarat**	96900	PPS sampling of clusters [all Rural cluster-village, Urban cluster-Urban ward in state in one list]	85 [R = 53, U = 32]	Interview, Digital Radiography		Conventional
**Faridabad**	90000	systematic sampling of clusters Stratified based on urban and rural [rural cluster = village, urban cluster = population residing in the jurisdiction of two adjacent polling booths]	36 [R = 12, U = 24]	Interview	i.Symptoms present ii. History of ATT	FM
**Kanpur**	90000 for both districts combined^^^	PPS sampling of clusters stratified by Rural /Urban [Rural cluster = village, Urban cluster = Urban enumeration block]	17 [R = 6, U = 11]			Conventional
**Banda**			35 [R = 32, U = 3]			Conventional
**Jabalpur**	90000	Simple Random Sampling of clusters stratified by Rural and Taluqs/Urban [Rural cluster = village, Urban cluster = Urban ward]	37 [R = 4, U = 33]			Conventional
**SAS Nagar**	90000	Simple Random Sampling of clusters stratified by Rural /Urban [Rural cluster = village, Urban cluster = Urban ward]	30 [R = 12, U = 18]			Conventional

PPS- probability proportionate to Size; R: Rural, U: Urban, MMR: Mass Miniature Radiography, ATT: Anti-TB Treatment, FM: Fluorescent Microscopy, Fixed cluster size in Chennai (600), Gujarat (1140), SAS Nagar (3000); entire cluster in other sites^;^

^#^In Nelamangala, sample size originally estimated at 47828 was revised after the X-ray equipment broke down mid-way.

^Sample size was estimated for Banda and Kanpur districts combined together with the intention to estimate the prevalence in two districts combined; however, the results are presented separately as sample size covered in each was found to be adequate to obtain precise estimates in individual districts

^$^Sputum smear examination using conventional microscope after straining by Zeihl-Neelsen method

### Sampling techniques (Table 2)

Simple random sampling was undertaken for selection of clusters in Nelamangala and Probability proportionate to size (PPS) sampling in Chennai. Simple random sampling of clusters stratified by rural and urban strata was adopted in Thiruvallur, SAS Nagar and Jabalpur. PPS sampling stratified by rural and urban strata was adopted in the remaining six sites. Villages were the rural clusters except in Nelamangala where each rural cluster corresponded to ‘Gram panchayat’ each consisting of 5–12 villages. Urban blocks/wards represented urban clusters except in Thiruvallur where each urban cluster corresponded to municipality/corporation/cantonment/notified town area; in Faridabad, each cluster corresponded to the population residing in the jurisdiction of two adjacent polling booths.

### Survey procedures

Survey procedures were carried out according to standard operating procedures specifically developed and pilot tested.

Fieldwork at each survey site was carried out by a trained team of enumerators, symptom elicitors, X-ray technicians where applicable, sputum collectors, planners and supervisors. The laboratory procedures and reading of X-ray films were performed by experienced personnel in the respective implementing institutes. All the field staff as well as the laboratory staff and X-ray readers were trained in survey methods uniformly at National Institute of Research in Tuberculosis, Chennai (NIRT).

Survey procedures involved the following activities:-

#### Planning

A planning visit was made to every cluster to familiarize local leaders and officials about the survey objectives and procedures and seek their cooperation. Thereafter, the planner went around the cluster, met groups of people and distributed printed material giving details of the survey in order to obtain their cooperation. He drew a rough sketch of the lanes and approximate number of houses within them, including hamlets if any. In each cluster, a suitable location for setting up survey center where mobile X-ray unit and spot sputum collection team would be stationed was arranged in consultation with the local leaders. A date and time to commence survey work was agreed upon mutually.

#### Enumeration and registration

On the appointed day, enumerators went to each household and explained about the purpose of the survey and procedures. They then queried either the head of the family or an elderly person regarding the number of people, age and sex of each individual and confirmed their residential status. Each eligible person (≥15 years of age, resident for ≥6 months) was registered into the survey on an individual card and allotted a unique identification study number (UID), after obtaining informed written consent. The necessary identification particulars were recorded. All households in the entire cluster were systematically covered.

#### Screening (Table 2)

In all the sites, a symptom elicitor interviewed each registered individual for presence of one or more of the following symptoms—persistent cough for ≥2 weeks, unexplained fever for ≥1 month, chest pain for ≥1 monthand history of passing blood in sputum any time in last 6 months. History of anti-TB treatment (ATT) -previous / current was also elicited from each individual. For quality control of symptom screening, 10% of eligible individuals were re-interviewed for presence of any of the symptoms as above and / or history of ATT.

Additional screening of registered individuals was undertaken by mobile chest radiography using digital unit in Gujarat and Mass Miniature Radiography (MMR) in Thiruvallur, Wardha and Chennai; in Nelamangala, only 36.8% of the registered individuals could be screened by MMR due to its breakdown mid-way through the survey. Pregnant women and bed ridden persons were excluded.

Each radiography image after processing in the respective institute was read independently by two X-ray readers.

#### Eligibility for sputum collection

Individuals having symptoms suggestive of PTB and / or history of ATT were eligible for sputum examination. Where screening by chest radiography was also carried out, individuals showing any pulmonary abnormality by any of the two readers were also eligible for sputum examination. In Wardha and Bangalore, participants with radiograph labeled as technically inadequate were also eligible for sputum examination. In Thiruvallur, Chennai and Gujarat, participants with technically inadequate were subjected to repeat chest radiography.

#### Sputum collection and transportation

At the survey center, a Laboratory Technician (LT) briefed the eligible individual on how to cough and bring out a good purulent sputum specimen and spit into a pre-numbered sterilized screw capped sputum bottle. The sputum collection was supervised by a lab attendant. After a satisfactory extraction of the spot specimen, the individual was given a pre-numbered empty bottle and was advised to similarly collect another specimen at home early morning next day. All bottles containing sputum specimens which had been marked with UID of the individuals were carefully kept in a tight sputum box and transported to the laboratory of the implementing institute on the day of collection in seven survey sites. In Banda and Kanpur, 1% Cetyl-Pyridinium-Chloride (CPC) was added to each container, packed using aseptic precautions and transported under cold chain to the laboratory within 72 hours, as per RNTCP guidelines^16^. In Gujarat, sputum specimens were transported similarly to the Intermediate Reference Laboratory.

#### Bacteriological examination

Each sputum specimen was subjected to smear and culture examinations.

All sputum processing was done in a Biosafety cabinet. Taking all aseptic precautions and using new labelled slides, two direct smears were made from each sputum specimen. At four sites, smears were stained with auramine for fluorescent microscopy while other sites performed Ziehl- Neelsen staining for microscopy under light binocular microscope.

After taking out the amount for smears, each specimen was homogenized and transferred to a McCartney bottle. For decontamination, 4% NaOH was added in a volume twice that of sputum specimen (Modified Petroff’s Method) and incubated in a shaker for 20 minutes. Sterile distilled water was then added up to the neck of the bottle and centrifuged at 3000g for 15 minutes. The supernatant was decanted and the deposit was inoculated onto two slopes of Lowenstein-Jensen (LJ) medium. Cultures were incubated at 37^0^ C and examined daily for first week every week for presence of Mycobacterial colonies for a period of 8 weeks. The culture was discarded in case of contamination or no growth at 8 weeks. If growth was observed, it was subjected to Niacin test and further incubated on LJ medium containing Para-nitro Benzoic acid (PNB) in a concentration of 500 μg/ml. If Niacin test was found to be positive and no growth was observed on PNB containing medium, the isolate was labeled as *Mycobacterium tuberculosis* (*M*. *tuberculosis*).

An individual was considered to be

- **Smear positive TB case** if the smear of either of the sputum specimen showed one or more acid fast bacilli (AFB), irrespective of culture result.
- **Culture positive TB case** if either of the specimens exhibited one or more colonies of *M*. *tuberculosis*, irrespective of smear result.
- **Bacteriologically positive TB case** if at least one of the two specimens was smear and/or culture positive.

### Ethical considerations

The survey protocols were approved by Ethics committees of the respective Institutes.

Benefits of participation in the survey in terms of ruling out / diagnosis of TB and subsequent treatment under RNTCP if required were explained to each individual by the field staff and also by using printed matter in local language and written consent sought for participation in the survey.

Individuals diagnosed with bacteriologically positive PTB and those with radiography image suggestive of PTB were referred to the nearest Government health facility for further action as per RNTCP guidelines.

### Statistical methods

At each site, data was digitalized by two independent data entry operators after checking and correcting the data collected on cards. The data so entered was matched and the errors rectified by re-entry of the records.

Analysis was undertaken uniformly for all the sites.

Prevalence was estimated after correcting for the bias introduced by incompleteness of data, using logistic regression model with robust standard error [15]. To include all eligible individuals in analysis, missing value imputation was undertaken for individuals: (a) without symptom screening (b) symptoms present but the result of one or both sputum specimen not available. This method accounted for clustering in survey design, variation in number of individuals tested in each cluster, between-cluster variability and uncertainty in estimating standard error, under the assumption that in sites where both screening methods were used, data were missing at random within groups of individuals belonging to same sex, age-group, TB symptoms and X-ray results. In sites where only screening by interview was used, data were assumed to be missing at random within groups of individuals belonging to same sex, age-group and TB symptoms. Model was then fitted with this particular variable as outcome variable and other variables as explanatory variables. This was done sequentially in the order of proportion of data that were missing starting with variables with least missing data. Finally, a logistic regression model with smear/culture/bacteriologically confirmed TB as the outcome variable and sex, age-group, TB symptoms and X-ray result as explanatory variables were fitted. Individual-level analyses were performed using logistic regression models, with and without multiple imputation. These were a) with robust standard errors based on observed between-cluster variability adjusting the SE but not the point estimates itself and b) random effects logistic regression adjusting both point estimates and their SEs was examined. Newly imputed values were used as starting values for next iteration of the process which was undertaken in ten cycles in order to obtain one imputed data set. Five such data sets were imputed and the average of their prevalence was taken as final prevalence. The overall point prevalence of TB and 95% confidence interval was calculated for each imputed dataset using random-effects logistic regression model.

Then an average of the estimates of TB prevalence from each of the imputed datasets was calculated, with a 95% confidence interval that takes into account both a) the variation in the estimate of point prevalence among imputed datasets and b) sampling variation including the effect of the clustering in the survey design [15]. The standard library functions provided in stata ver 12 were called to perform the random effect model analysis to adjust both the point estimates and their SEs.

From each site where both screening tools–interview, chest X-ray were used, the weighted correction factor for prevalence due to non-screening by X-ray was estimated, the weight being equal to inverse of the variance; for Nelamangala, data only from clusters where both tools were employed was used for this purpose. For sites using screening by interview only, prevalence was corrected for non-screening by chest radiography on applying this correction factor estimated at 1.31.

Chi-square test with continuity correction was used to test the significance of differences between proportions and p-values<0.05 were considered significant.

Cochran’s Q test was applied to test the heterogeneity between the estimated prevalence at different sites. Since significant heterogeneity was revealed by the test, pooled estimate of prevalence was obtained using Random Effects Model after excluding data from SAS Nagar where the estimated prevalence was found to be inexplicably low.

Prevalence of all forms of TB in the entire population (all age groups) was estimated by adjusting the pooled prevalence of bacteriologically positive PTB for extra pulmonary TB (EPTB) in adults considering its proportion at 15% (based on RNTCP data) and for all types of TB in pediatric age group considering its proportion at 9% and 30% of the population being in pediatric age group [16, 18, 19].

## Results

**Fig 1:**
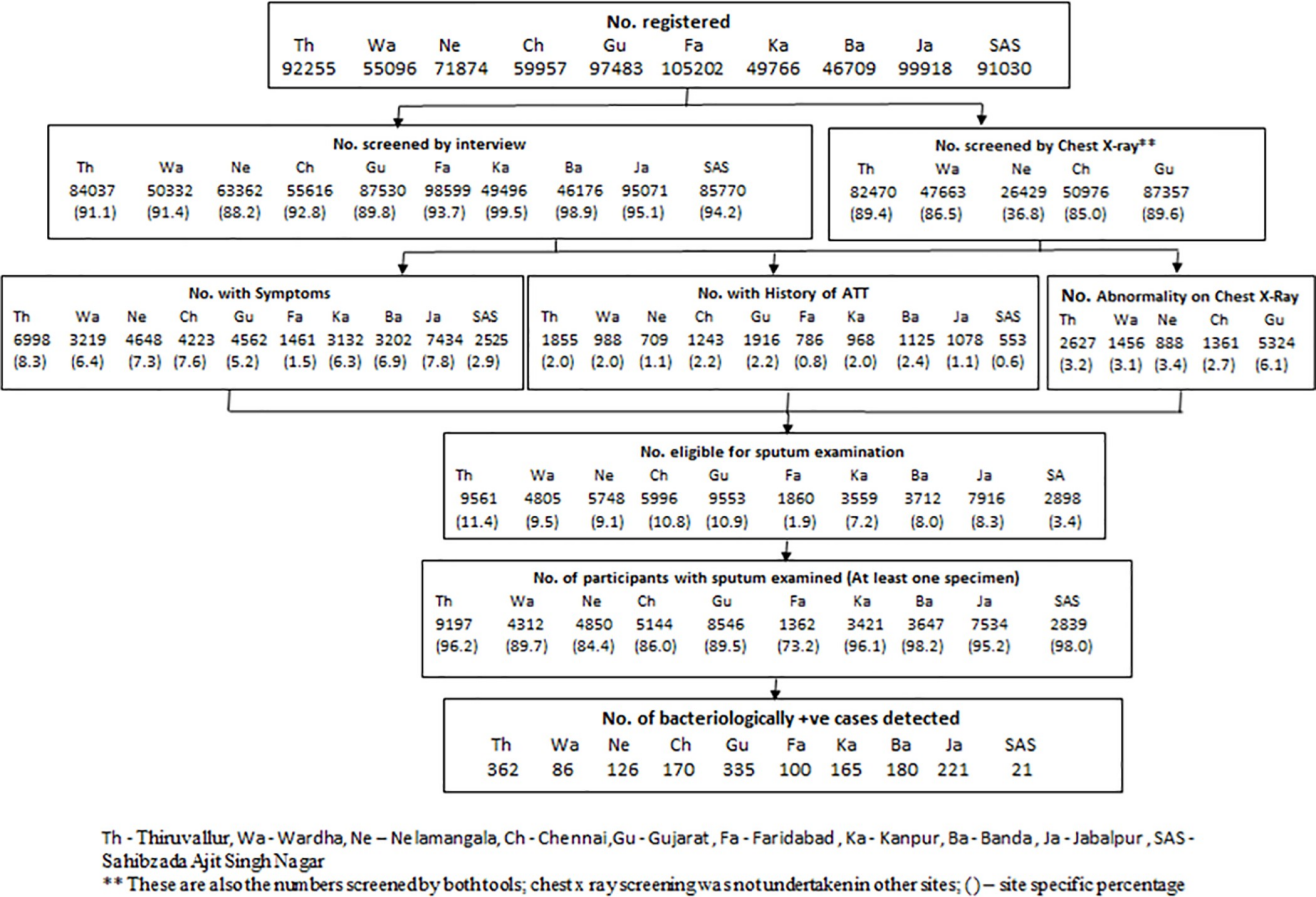
Flowchart depicting numbers of individuals registered, numbers screened by interview and/or Chest X-ray, numbers eligible for sputum examination, numbers actually examined and the numbers of bacteriologically confirmed pulmonary TB cases detected by site.

The flow chart given in Fig 1 summarizes the numbers of individuals registered in individual sites, numbers screened by interview and chest X-ray, proportions having symptoms, h/o ATT and abnormality on CXR, sputum examination-number elligible based on screening, number actually examined and the detected bacteriologically confirmed PTB cases.

### Study population

Number of individuals registered varied between 46709–105202 in different sites and the proportion screened by interview between 88.2–99.5%. In the sites where screening by chest radiography was carried out, the proportion screened by radiography varied between 36.8–89.6%.

### Eligible for sputum examination

The proportion with symptoms and history of ATT varied between 1.5–8.3% and 0.6–2.4% respectively. The proportion with pulmonary abnormality on radiograph varied between 2.7–6.1% out of those screened by radiography. Altogether, in four sites where screening by both interview and radiography were undertaken in the entire study sample, the proportion eligible for sputum examination varied between 9.5–11.4%. In Nelamangala, where screening by radiography was undertaken only in 36.8% of the individuals, the proportion eligible for sputum examination was 9.1%. In sites where screening was undertaken only by interview, the proportion eligible varied between 1.9–8.3%. Of those eligible, at least one sputum specimen was collected and examined varied between 73.2–98.2%.

### Cases detected

Number of bacteriologically positive cases detected during the surveys varied between 21–362 in different sites; distribution by smear and culture results is given in Table 3.

**Table 3:** Distribution of cases detected during surveys by smear and culture result.

Site	No. of bacteriologically positive cases	S+C+	S+C-	S-C+	S+C Contaminated	Proportion of culture positive cases out of all bacteriological positive cases	Proportion (%) smear positive out of culture positive	Proportion (%) culture positive out of smear positive
**Sub division of Thiruvallur district**	362	125	29	207	1	91.7	37.7	80.6
**Wardha**	86	35	18	31	2	76.7	53.0	63.6
**Nelamangala sub-division of Bangalore Rural district**	126	28	28	69	1	77.0	28.9	49.1
**Chennai city**	170	67	67	59	3	74.1	53.2	48.9
**Gujarat**	335	140	83	101	11	71.9	58.1	59.8
**Faridabad**	100	63	9	19	9	82.0	76.8	77.8
**Kanpur**	165	65	81	12	7	46.7	84.4	42.5
**Banda**	180	54	46	69	11	68.3	43.9	48.6
**Jabalpur**	221	108	39	73	1	81.9	59.7	73.0
**SAS Nagar**	21	4	0	17	0	100.0	19.0	100.0

S: Smear, C: Culture, +: Positive, -: Negative

The proportion of culture positive out of all bacterioloogically positive cases varied widely between 46.7–91.7%; it was 100% in SAS Nagar which is the outlier due to small number of detected cases.

Proportion of smear positive out of culture positive cases varied widely between 28.9–84.4%; the lowest proportions were observed in Thiruvallur and Nelamangala.

With the proportions of culture positive out of smear positive cases varying between 42.5–80.6% across sites, there were a significant proportion of smear positive cases found to be culture negative.

Of the cases that would be detected if screening was restricted to interview only, the proportion having cough with or without other symptoms varied between 65.9–92.1%. The proportion having other symptoms but not cough varied 1.5–18.6% and the proportion having h/o ATT but no symptom between 3.3–21.3% across different sites; the increase in case yield by chest radiography over and above the cases detected by interview varied between 31.4–65.9% across four sites; it was lower in Nelamangala due to only 36.8% of study sample screened by X-ray (Table 4).

**Table 4:** Distribution of bacteriologically positive cases detected during surveys by symptom, history of ATT and chest radiography.

Site	No. of cases detected having any symptom or h/o ATT	No. having Cough with or without other symptoms or h/o ATT	No. with symptoms other than cough or h/o ATT	Additional cases detected by screening for H/o ATT	Additional cases detected by Chest X-ray screening
**Sub division of Thiruvallur district**	235	155 (65.9)	30 (12.7)	50 (21.3)	127 (54.0)
**Wardha**	59	41 (69.5)	11 (18.6)	7 (11.8)	27 (65.9)
**Nelamangala sub-division of Bangalore Rural district**	110	89 (80.9)	12 (10.9)	9 (8.2)	16 (14.5) ^
**Chennai city**	119	85 (71.4)	14 (11.7)	20 (16.8)	51 (42.8)
**Gujarat**	255	223 (87.5)	4 (1.5)	28 (10.9)	80 (31.4)
**Faridabad**	100	84 (84.0)	5 (5.0)	11 (11.0)	NA
**Kanpur**	165	141 (85.5)	11 (6.6)	13 (7.8)	
**Banda**	180	166 (92.1)	8 (4.4)	6 (3.3)	
**Jabalpur**	221	195 (88.2)	13 (5.8)	13 (5.8)	
**SAS Nagar**	21	17 (80.9)	1 (4.7)	3 (14.3)	

(): Percentages

NA: Not Applicable

^Chest X-ray screening was undertaken in only 36.8% of eligible population

### Prevalence of bacteriologically confirmed pulmonary TB by site

**Fig 2:**
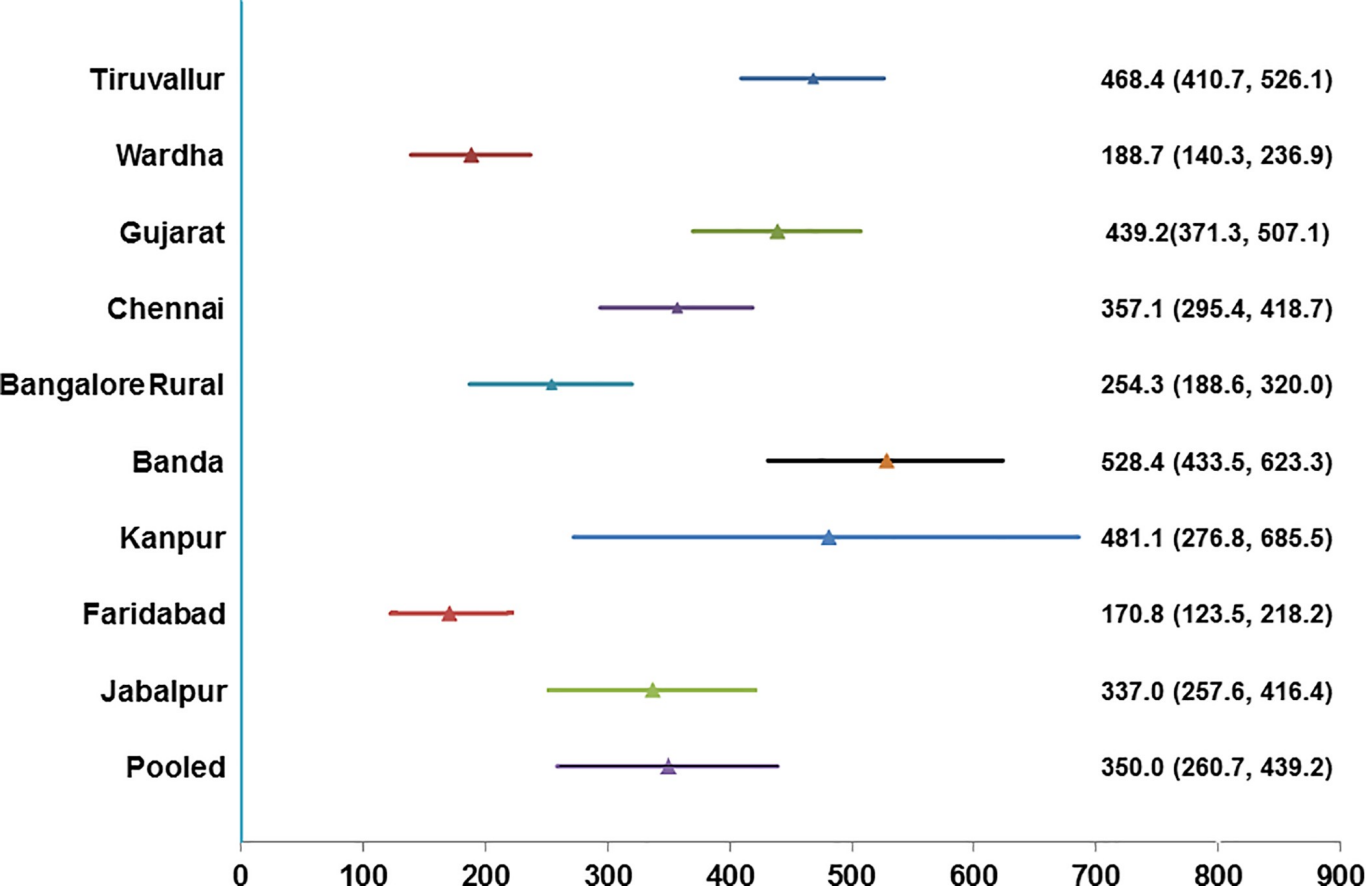
Estimated prevalence of bacteriologically positive pulmonary TB by site and the pooled prevalence.

Excluding SAS Nagar, estimated prevalence of bacteriologically positive PTB varied between 170.8–528.4 per 100 000 populations in different sites, being the highest in the two districts of Uttar Pradesh state and the minimum in northern districts of SAS Nagar and Faridabad-the districts with highest notification rates among the ten sites. (Table 1, Table 5, Fig 2). Estimated prevalence of smear positive PTB and culture positive PTB varied between 108.4–428.1 and 147.9–429.8 per 100 000 populations respectively across different sites.

**Table 5:** Estimated prevalence of smear positive, culture positive and bacteriologically positive pulmonary TB by survey site.

Site	Prevalence per 100 000 populations		
	Smear positive PTB	Culture positive PTB	Bacteriologically positive PTB
**Sub division of Thiruvallur district**	197.7 (161.8, 233.5)	429.8 (375.9, 483.8)	468.4 (410.7, 526.7)
**Wardha**	121.1 (84.8, 157.3)	147.9 (101.5, 194.3)	188.7 (140.3, 236.9)
**Nelamangala sub-division of Bangalore Rural district**	108.4 (74.6, 142.2)	194.9 (133.2, 256.5)	254.3 (188.6, 320.0)
**Chennai city**	224.9 (174.2, 273.2)	262.1 (216.0, 308.2)	357.1 (295.4, 418.7)
**Gujarat**	285.3 (226.0, 344.7)	312.2 (255.0, 369.4)	439.2 (371.3, 507.1)
**Faridabad**^Ω^	139.0 (98.3,179.7)	154.0 (113.8, 194.1)	170.8 (123.5, 218.2)
**Kanpur**^Ω^	428.1 (263.1, 592.9)	220.6 (115.3, 326.0)	481.1 (276.8, 685.5)
**Banda**^Ω^	323.3 (243.4, 403.3)	366.8 (307.6, 426.1)	528.4 (433.5, 623.3)
**Jabalpur**^Ω^	226.9 (163.3, 290.5)	276.5 (216.3, 336.8)	337.0 (257.6, 416.4)
**SAS Nagar**^Ω^	8.2 (0.5, 15.8)	24.1 (12.8, 35.4)	31.8 (16.9, 46.8)
**Pooled estimate** **(excluding SAS Nagar)**	216.3 (165.8, 266.8)	262.8 (196.0, 329.6)	350.0 (260.7, 439.2)

PTB: Pulmonary TB

Ω: Prevalence in these sites has been corrected for non–screening by X-ray

(): 95% Confidence Interval

### Prevalence by stratum, sex and age group

Excluding SAS Nagar, prevalence of bacteriologically confirmed pulmonary TB varied across sites in urban areas between 126.8–389.0 and in rural areas between 189.1–810.9 per 100,000 populations. It was more in rural than the urban areas in all the seven sites where study population included participants both from rural and urban areas. While the pooled ratio of this difference was two, it varied from a highest of 2.1 in Kanpur to the lowest of 1.2 in Wardha.

Prevalence in females and males varied between 73.9–349.4 and 239.3–800.8 respectively across different sites. While the pooled ratio in males to female prevalence was three it varied between1.7–6.0 across sites, being the highest in Nelamangala and the lowest in Kanpur (Table 6).

**Table 6:** Estimated prevalence of bacteriologically positive PTB per 100 000 populations by stratum and survey site.

Site	By stratum			By sex		
	Urban	Rural	Ratio	Female	Male	Ratio
**Sub division of Thiruvallur district**	239.3 (98.2, 380.4)	496.6 (438.8, 554.4)	1:2.1	154.0 (107.5, 200.5)	800.8 (687.6, 914.0)	1:5.2
**Wardha**	153.8 (80.9, 226.7)	189.1 (122.6, 255.6)	1:1.2	106.5 (60.5, 152.5)	247.9 (174.5, 321.3)	1:2.3
**Nelamangala sub-division of Bangalore Rural district**	NA	254.3 (188.6, 320.0)	NA	73.9 (34.6, 113.3)	439.8 (308.8, 570.8)	1:6.0
**Chennai city**	357.1 (295.6, 418.6)	NA	NA	139.0 (87.5, 190.5)	583.2 (467.4, 699.0)	1:4.2
**Gujarat**	389.0 (298.2, 479.8)	487.3 (383.2, 591.4)	1:1.3	248.8 (186.7, 311.0)	610.9 (512.0, 709.9)	1:2.5
**Faridabad**^Ω^	126.8 (74.6, 179.0)	253.4 (129.1, 377.7)	1:2.0	110.4 (72.6, 148.3)	239.3 (161.2, 317.4)	1:2.2
**Kanpur**^Ω^	328.8 (242.3, 415.3)	810.9 (539.3, 082.5)	1:2.5	349.4 (210.7, 488.2)	591.4 (326.0, 856.8)	1:1.7
**Banda**^Ω^	380.1 (202.1, 558.1)	580.8 (479.8, 681.8)	1:1.5	346.5 (242.7, 450.4)	712.5 (576.9, 848.2)	1:2.1
**Jabalpur**^Ω^	232.6 (174.6, 290.5)	486.0 (361.9, 610.1)	1:2.1	153.7 (112.0, 195.3)	507.8 (378.4, 637.2)	1:3.3
**SAS Nagar**^Ω^	21.8 (0, 51.5)	49.8 (22.8, 76.8)	1:2.3	20.1 (3.4, 36.7)	49.1 (24.9, 73.4)	1:2.5
**Pooled estimate (excluding SAS Nagar)**	265.8 (190.5, 341.0)	425.7 (305.4, 545.9)	1:1.6	168.2 (123.5, 212.8)	521.5 (375.4, 667.6)	1:3.1

Ω: Prevalence in these sites has been corrected for non–screening by X-ray

(): 95% Confidence Interval

NA: Not Applicable since the study population belonged to only one type of area.

Prevalence increased with age group across all the sites (data not presented in tables).

### Pooled prevalence

Pooled estimate of prevalence of bacteriologically positive PTB was 350.0 (260.7, 439.2). Pooled prevalence of smear positive and culture positive PTB was 216.3 (165.8, 266.8) and 262.8 (196.0, 329.6) respectively.

Pooled estimate in urban and rural strata were 265.8 (190.5, 341.0) and 425.7 (305.4, 545.9) respectively. Pooled estimate in females and males were 168.2 (123.5, 212.8) and 521.5 (375.4, 667.6) respectively.

The overall prevalence of TB (all ages, all types) was estimated at 300.7 (223.7–377.5) per 100000 population; this would most closely apply to the year 2009, the mid-year of above mentioned surveys.

## Discussion

Prevalence of bacteriologically positive PTB in adults was found to vary from 170.8 (123.5, 218.2) to 528.4 (433.5, 623.3) per 100 000 populations in different sites and prevalence of smear positive PTB from 108.4 (74.6, 142.2) to 428.1 (263.1, 592.9), excluding the results from SAS Nagar. This wide variation in prevalence indicates diversity in TB burden in different parts of the country. Though the lowest prevalence rates were obtained in sites with highest all TB notification rates, no clear correlation was seen between prevalence and notification or treatment success rates. These differences in prevalence may be attributed to a mix of efficiency of TB control interventions, living conditions and local cultural factors.

Pooled prevalence of bacteriologically positive PTB in adults was 350.0 (260.7, 439.2). On correcting for burden of extra-pulmonary TB in adults and TB in children, the overall prevalence of TB (all ages, all types) was estimated at 300.7 (223.7–377.5) per 100,000 populations. This estimate would most closely correspond to the year 2009 which is the mid-year of the nine surveys considered for obtaining the pooled estimate. Our pooled estimate is lower than the prevalence considered nationally by WHO at 390/100 000 based on prevalence in Gujarat corrected for extra pulmonary and pediatric TB [1]. Our pooled estimate is likely to be biased towards underestimation due to non-screening by radiography in half the sites and using MMR in four sites, which is less sensitive than digital radiography [20]. Even though we corrected for non-screening by radiography, the proportion of participants with symptoms was low especially in Faridabad where screening was done only by interview; the overall prevalence would depend upon the quality of symptoms screening.

As in the present surveys, similar variation in prevalence of bacteriologically positive PTB was observed during surveys carried out previously [4, 5]. During the nation-wide survey in 1955–58 when about 300,000 persons aged ≥5 years residing in a sample of 150 villages, 6 cities and 30 towns in six hypothetically formed zones were investigated; prevalence varied between 200–800 per 100 000 populations in different parts of the country[3]. Since screening was undertaken only by MMR and one sputum specimen and a laryngeal swab were examined by microscopy (Z-N) and culture (L-J), the national level estimate of 400 per 100,000 populations obtained during that survey cannot be compared to our pooled estimate. Prevalence of bacteriologically positive PTB among adults varied between 200–1100 per 100,000 population during various surveys carried out in different parts of the country between 1958–2005.

Trends in prevalence could be ascertained from three of the present survey sites. In Thiruvallur, four serial surveys carried out between 1999–2008 revealed overall decline of 35% in bacteriologically positive PTB from the 1^st^to 4^th^ survey; RNTCP in this area was implemented from 1999 [7]. In Nelamangala, a survey had been conducted in 1975 using similar screening technique as during the present survey and the crude prevalence (Number of cases detected ÷ Number screened by interview and if eligible for sputum examination results of microscopy and culture available for both sputum specimen) of culture positive PTB was found to be 311 per 100,000 populations [21]. Crude prevalence of culture positive TB in the present survey was 137 per 100000 populations implying an overall decline of 56% between 1975 and 2009. In Wardha, a district wide survey was carried out during 1982–88 in which using screening by interview only, the crude prevalence of bacteriologically positive TB was estimated at 229.2 per 100,000 population in ≥15 years age-group [22]. In the present survey in Wardha, crude prevalence of bacteriologically positive TB ignoring screening by MMR was estimated at 117.2 per 100,000 population implying a decline of 49% between 1985 and 2008–the mid-years of respective surveys [8, 22]. RNTCP in Nelamangala and Wardha is being implemented from 2002. Serial surveys carried out in specific areas before implementation of RNTCP had revealed no significant change in prevalence [4, 5, 23–25]. Therefore the declining trend in prevalence as observed in Thiruvallur, Nelamangala and Wardha may be attributable to RNTCP implementation.

In our surveys, prevalence was significantly higher in rural compared to urban areas across all sites contrary to the findings of the nation-wide survey in 1955–58 [3]. Lower prevalence in urban areas in present times could be attributable to better access to health care as well as better living conditions compared to the rural areas. It is estimated that about 85% of the medical professionals in the private sector are mostly located in the urban areas [26].

Prevalence was significantly higher in males compared to females and the ratio varied from 1.7 to 6.0 across different sites. Prevalence increased with age, as also observed during previously conducted surveys.

Of all symptomatic cases detected during our surveys, cough≥2 weeks was present in 65.9–92.1% across different sites. The surveys revealed additional yield varying between 1.5–18.6% by screening for other symptoms suggestive of PTB. A significant additional yield (3.3–21.3%) of cases was also observed by screening for history of ATT which is not listed as one of the screening criteria in the WHO guide [15]. Relatively lower proportion of smear positive out of all culture positive cases in Nelamangala and Thiruvallur might be related to more thorough screening in these areas leading to early detection of cases. The proportion of culture positive out of all smear positive cases varied between 42.5–80.6%. Low proportions of culture positive cases in some sites could be attributed to either the presence of dead bacilli or absence of growth even in the event of live bacilli which may fail to grow in-vitro if the patient was on ATT with rifampicin containing regimen [27].

A national level survey in a representative sample of the population using screening by interview as well as digital radiography is presently in planning phase.

Results of the present surveys reveal that India continues to be a high TB burden country. Translating our pooled estimate to the entire country’s population, there were 3.5 million prevalent TB cases in the year 2009. The observed ratio of our pooled prevalence of smear positive to notification rate of smear positive (new + previously treated) cases under RNTCP at the national level in the year 2009 was found to be 3:1; this ratio varied between 1.9–7.2 in individual survey sites (Table 7). This suggests the need for drastic improvements in case finding efficiency including in the private sector. Universal access for diagnosis of drug sensitive as well as drug resistant TB, reduction in diagnostic delay by increasing community awareness amongst all target groups, provision of more sensitive diagnostic tools including in the private sectors and further improvement in treatment success rates of drug sensitive as well as drug resistant TB are warranted in order to reduce the TB burden in the country.

**Table 7:** Prevalence to notification ratio.

Site	Estimated prevalence of smear positive cases	RNTCP Notification rate of smear positive cases for corresponding mid-year of the survey	Prevalence to notification ratio
**Wardha**	121.1	62 (2008)	2.0
**Chennai city**	224.9	70 (2011)	3.2
**Gujarat**	285.3	95 (2011)	3.0
**Faridabad**	139	74 (2008)	1.9
**Kanpur**	428.1	59 (2009)	7.3
**Banda**	323.3	76 (2009)	4.3
**Jabalpur**	226.9	69 (2009)	3.3
**SAS Nagar**	8.2	93 (2008)	0.1
**Pooled estimate (excluding SAS Nagar)**	216.3	72* (2009)	3.0

Thiruvallur and Nelamangla not included in this table since notification rates are not disseminated in surveillance reports for sub-district levels. New + previously treated; *National level

## Supporting information

S1 File DPS final data. The zipped file folder contains 10 SPSS dataset files with their file names representing the survey sites.
(RAR)

## References

[1] World Health Organization. Global tuberculosis Report 2016 World Health Organization 2016. WHO press Geneva. WHO/HTM/TB/2016.13.

[2] Murray CJ, Ortblad KF, Guinovart C, Lim SS, Wolock TM, Roberts DA, et al Global, regional, and national incidence and mortality for HIV, tuberculosis, and malaria during 1990–2013: a systematic analysis for the Global Burden of Disease Study. Lancet 2014; 384:1005–70. 10.1016/S0140-6736(14)60844-8 25059949 PMC4202387

[3] Indian Council of Medical Research. Tuberculosis in India: A National Sample Survey; ICMR special report series No.34, 1955–1958, 1959. ICMR, New Delhi.

[4] Chadha V K. Tuberculosis Epidemiology in India: a review. Int J Tuberc Lung Dis. 2005; 9(10): 1072–1082. 16229217

[5] Chadha VK. Epidemiology of pulmonary tuberculosis In: Textbook of pulmonary and critical care medicine 2011, first edition Jaypee Brothers medical publishers (P) Ltd, New Delhi, p.489–510.

[6] Chadha VK, Kumar P, Anjinappa SM, Singh S, Somashekar N., Joshi M. V., et al Prevalence of Pulmonary Tuberculosis among Adults in a Rural Sub-District of South India. PLoS ONE 2012; 7(8): e42625 10.1371/journal.pone.0042625 22956993 PMC3431961

[7] Kolappan C, Subramani R, Radhakrishna S, Santha T, Wares F, Baskaran D, et al Trends in the prevalence of pulmonary tuberculosis over a Period of seven and half years in a rural community in South India with DOTS. Indian J Tuberc 2013; 60:168–176. 24000495

[8] Narang P, Mendiratta DK, Tyagi NK, Jajoo UN, Tayade AT, Parihar PH, et al Prevalence of pulmonary tuberculosis in Wardha district of Maharashtra, Central India. J Epidemiol Glob Health. 2015 4 29 pii: S2210-6006(15)00035-0. 10.1016/j.jegh.2015.03.002 25936795 PMC7325829

[9] Rao VG, Bhat J, Yadav R, Gopalan GP, Nagamiah S, Bhondeley MK, et al (2012) Prevalence of Pulmonary Tuberculosis—A Baseline Survey In Central India. PLoS ONE 7(8): e43225 10.1371/journal.pone.0043225 22952651 PMC3430677

[10] Aggarwal AN, Gupta D, Agarwal R, Sethi S, Thakur JS, Anjinappa SM, et al Prevalence of pulmonary tuberculosis among adults in a north Indian district. PLoS One. 2015 2 19; 10(2):e0117363 10.1371/journal.pone.0117363 eCollection 2015. 25695761 PMC4335010

[11] Katoch K, Chauhan DS, Yadav VK, Katoch V, Upadhay P Sharada MA, et al (2015) Prevalence Survey of Bacillary Pulmonary Tuberculosis in Western Uttar Pradesh, India. J Infect Pulm Dis 1(2): 10.16966/jipd.108

[12] Sharma SK, Goel A, Gupta SK, Mohan K, Sreenivas V, Rai SK, et al Prevalence of tuberculosis in Faridabad district, Haryana State, India. Indian J Med Res. 2015; 141(2): 228–235. 25900959 10.4103/0971-5916.155593 PMC4418160

[13] State TB Cell, Department of Health & Family Welfare, Government of Gujarat. Report on Population based survey to assess prevalence of pulmonary tuberculosis cases in the state of Gujarat, India (2011–12).December 2013.

[14] Dhanaraj B, Papanna MK, Adinarayanan S, Vedachalam C, Sundaram V, Shanmugam S, et al (2015) Prevalence and Risk Factors for Adult Pulmonary Tuberculosis in a Metropolitan City of South India. PLoS ONE 10(4): e0124260 10.1371/journal.pone.0124260 25905900 PMC4408069

[15] World Health Organization. Tuberculosis prevalence surveys:a handbook World Health Organization, Geneva 2011;WHO/HTM/TB /2010.17.

[16] Central TB Division, Directorate General Health Services, Ministry of Health and Family Welfare, Government of India- Revised National TB Control Programme Annual Status Reports. Available at http://www.tbcindia.nic.in. Accessed 2016 August 30.

[17] Central TB Division, Directorate General Health Services, Ministry of Health and Family Welfare, Government of India- Revised National TB Control Programme, Training Manual for Mycobacterium tuberculosis Culture & Drug susceptibility testing. Available at http://www.tbcindia.nic.in. Accessed 2017 January 03.

[18] Dodd PJ, Gardiner E, Coghlan R, Seddon JA. Burden of childhood tuberculosis in 22 high-burden countries: a mathematical modelling study. The lancet global health, 2014; 2(8):453–458. 10.1016/S2214-109X(14)70245-1 25103518

[19] Office of the Registrar General & Census Commissioner, India. Projected Total Population by sex as on 1st March-2001-2026 India, States and Union Territories. URL: http://censusindia.gov.in/Census_Data_2001/Projected_Population/Projected_Population.pdf Accessed on 31st August 2016.

[20] van’t Hoog AH, Meme HK, Laserson KF, Agaya JA, Muchiri BG, Githui WA, et al Screening Strategies for Tuberculosis Prevalence Surveys: The Value of Chest Radiography and Symptoms. PLoS ONE 2012 7(7): e38691 10.1371/journal.pone.0038691 22792158 PMC3391193

[21] Gothi GD, Radha Narayan, Nair SS, Chakraborti AK, Srikantaramu N. Estimation of prevalence of bacillary TB on the basis of chest X-ray and symptomatic screening. Indian J Med Res 1976; 64: 1150–1159. 1086830

[22] National Tuberculosis Institute, Bangalore. Tuberculosis in a rural population of South India: a five-year epidemiological study. Bull World Health Organ 1974; 51(5):473–88. 4549498 PMC2366313

[23] Nayar S, Narang P, Tyagi NK. Field trial of short term intermittent chemotherapy against Tuberculosis Department of Community Medicine, Medicine & Microbiology, Mahatma Gandhi Institute of Medical Sciences, Sevagram, Wardha, Maharashtra, 1982–1989. Project [No.5/8/5/2/81/ECD-1 ID No-8102650], 1989. Report submitted to Indian Council of Medical Research, New Delhi.

[24] New Delhi TB Centre. Study of epidemiology of Tuberculosis in an urban population of Delhi-report on 30 year follow-up. Indian J Tuberc 1999; 46: 113–24.

[25] Tuberculosis Research Centre. Trends in the prevalence and incidence of tuberculosis in South India. International Journal of Tuberculosis and Lung Disease 2001; 5:142–57. 11258508

[26] Loh L C, Ugarte-Gil C, Darko K. Private sector contributions and their effect on physician emigration in the developing world. Bulletin of the World Health Organization 2013; 91:227–233. 10.2471/BLT.12.110791 23476095 PMC3590622

[27] Lorian V, Finland M. In vitro effect of rifampin on mycobacteria.AppliedMicrobiol 1969; 17(2):202–7. 10.1128/am.17.2.202-207.1969 PMC377649 4975656

